# ChemsexPH: The association between chemsex, HIV status and adherence to antiretroviral therapy among men who have sex with men in the Philippines

**DOI:** 10.1002/jia2.26323

**Published:** 2024-07-09

**Authors:** Rodenie Arnaiz Olete, Carol Strong, Katerina Leyritana, Adam Bourne, Nai‐Ying Monica Ko

**Affiliations:** ^1^ Department of Public Health College of Medicine National Cheng Kung University Tainan Taiwan; ^2^ Sustained Health Initiatives of the Philippines Mandaluyong City Philippines; ^3^ Australia Research Centre in Sex Health and Society La Trobe University Melbourne Victoria Australia; ^4^ Kirby Institute UNSW Sydney Sydney New South Wales Australia; ^5^ Department of Nursing, College of Medicine National Cheng Kung University Tainan Taiwan

**Keywords:** drug use, men who have sex with men, PrEP, Philippines

## Abstract

**Introduction:**

Chemsex, the use of psychotropic drugs before or during sexual intercourse, is associated with various HIV risk factors, including condomless sex and reduced adherence to pre‐exposure prophylaxis or antiretroviral therapy (ART). In the Philippines, there are still limited studies exploring the associations between chemsex, HIV status and ART adherence. This study aims to compare recent and lifetime chemsex engagement in association with self‐reported HIV status among Filipino men who have sex with men (MSM). We further explored the association between chemsex and ART adherence among people living with HIV engaged in chemsex.

**Methods:**

A cross‐sectional online survey of 479 Filipino MSM was conducted from 3 August to 1 December 2019. Demographic profiles, sexual behaviours, drug use, history of sexually transmitted infections (STIs), chemsex engagement and HIV status were collected and analysed. Bivariable and multivariable logistic regression were employed to assess the association between self‐reported HIV status and chemsex engagement.

**Results:**

Among the 479 respondents, Filipino MSM engaged in drug use and chemsex were generally older compared to those not engaged in drug use and chemsex (average age 31−33 vs. 29 years old; *p*<0.05). Methamphetamine was the most common drug for people who reported using drugs. An HIV‐positive status was associated with recent chemsex engagement (aOR = 5.18, *p*<0.05) and a history of STIs (aOR = 2.09, *p*<0.05). The subgroup analysis showed that 79% (166/200) of persons living with HIV were adherent to ART. There was no significant association found between chemsex and ART adherence in the logistic regression analyses.

**Conclusions:**

Chemsex behaviour, particularly recent chemsex engagement, is significantly associated with self‐reported HIV status. The emerging data on MSM engaged in chemsex require integration of a more person‐centred, comprehensive and robust harm reduction programmes into the existing combination prevention strategies in the country. Health education for Filipino MSM engaged in chemsex should prioritize raising awareness about methamphetamine effects and overdose risks, alongside proper medical management.

## 1 INTRODUCTION

The Philippines had a 237% increase in new HIV cases between 2010 and 2018, making it one of the countries with the highest HIV incidence in Southeast Asia [1, 2]. The majority of reported HIV cases in the Philippines are among men who have sex with men (MSM), with only 3% linked to people sharing non‐sterile needles [3]. Consequently, the HIV prevention efforts have been focused heavily on male‐to‐male sexual transmission risk. However, limited harm reduction programmes exist for Filipino people who inject drugs (PWID), especially for MSM engaged in chemsex—an emerging phenomenon in the Philippines.

Chemsex engagement involves psychotropic drug use to enhance sexual experiences [4, 5] and the subculture of “slamsex” involves intravenous drug injection—both often influenced by sensation‐seeking behaviours [6, 7] and pose increased risks of bloodborne virus transmission [8]. Common substances used in chemsex include crystal methamphetamine (“shabu” in the Philippines) and gamma hydroxybutyrate [9, 10, 11].

Both HIV and sexually transmitted infections (STIs) among MSM involved in chemsex are frequently associated with factors such as slamsex‐related needle‐sharing [6, 12, 13, 14, 15], condomless anal sex (CAS) [16, 17, 18], non‐adherence to antiretroviral therapy (ART) [18, 19, 20, 21] and decision‐making disinhibition leading to risk‐taking behaviours [7, 22, 23]. However, there are still limited studies clarifying the complex associations between chemsex, HIV and ART adherence. In the Philippines, ART accessibility has improved yet enrolment and treatment retention remains a challenge [24]. A facility‐based study conducted in Metro Manila found that people living with HIV (PLHIV) engaged in both injecting drug use and condomless sex exhibited lower ART adherence [24].

Dearth in Philippine‐based chemsex‐related research remains despite global evidence linking chemsex to HIV incidence among MSM [5, 26, 27]. A comprehensive review in Asia‐Pacific in 2010−2021 did not identify any recent Philippine‐based chemsex study [28]. Hence, this study aims to address this gap by comparing recent and historical chemsex engagement among Filipino MSM and determine its association with self‐reported HIV status. Additionally, a subgroup analysis was conducted to assess the association of chemsex engagement with ART adherence among Filipino PLHIV. This study underscores the necessity for country‐specific data in the Philippines to inform tailored prevention and harm reduction approaches, cautioning against solely relying on findings from other countries.

## 2 METHODS

An anonymous cross‐sectional online survey was conducted between 3 August and 1 December 2019, using voluntary response sampling posted on social media (i.e. Facebook, Twitter/X), consisting of four main parts: informed consent (placed at the first part of the online survey where the nature and purpose of the study were explained and ensuring confidentiality) and inclusion criteria screening, demographic profile, sexual health status including STI and HIV information, and drug use and chemsex experiences. Then, respondents were screened based on the following inclusion criteria: (1) Filipino citizen living in the Philippines at least within the last 6 months at the time of data gathering; (2) 18 years old and above; and (3) identifies as cisgender man having sex with men. The survey was voluntary and non‐incentivized, with the approval of the Philippine Health Research Ethics Board (20190715‐108‐NEC).

## 3 VARIABLES

### 3.1 Dependent variables

#### 3.1.1 Self‐reported HIV status

The dependent variable in this study was self‐reported HIV status. Respondents were asked to report their HIV status based on their most recent HIV testing. The responses were categorized into three groups: HIV positive, HIV negative and unknown status.

#### 3.1.2 ART adherence

For respondents who reported an HIV‐positive status, a visual analogue scale (VAS) was used to ask their adherence to ART, representing 0−100% adherence. Categories were either “adherent” (≥90%) or “non‐adherent” (<90%). The cutoff for adherence was set at 90% based on the previous study [29].

### 3.2 Independent variable

#### 3.2.1 Chemsex engagement

The independent variable of interest was chemsex engagement asked by *“When was the last time you used drugs while having sex?”* with answers corresponding to four groups of MSM: (a) non‐C/DU, representing non‐engagement in drug use or chemsex; (b) DU, indicating experience in drug use but not for chemsex; (c) CDU, indicating chemsex engagement but not recently; and (d) CDU6, indicating recent chemsex engagement within the last 6 months.

### 3.3 Covariates

#### 3.3.1 Demographic profile

This included measures such as age in years, location in the Philippines (categorized as Metro Manila/Luzon or the rest of the Philippines), education level (high school or lower vs. college or higher) and employment status. Location is a covariate because residing in major cities like Manila and Luzon is associated with higher accessibility to illicit drugs and HIV care facilities.

#### 3.3.2 Access to ART, and access to pre‐exposure prophylaxis

This measured access to antiretroviral medications after they responded to the HIV status question wherein HIV‐negative respondents were directed to questions on access to pre‐exposure prophylaxis (PrEP), while respondents who reported and HIV‐positive status were directed to questions on access to ART.

#### 3.3.3 Condomless anal sex

This measured the occurrence of penetrative anal or vaginal sex without using a condom, categorized as “≤6 months ago” or “>6 months or never.”

#### 3.3.4 History of STIs

Respondents were asked *“Have you ever been diagnosed with any STIs other than HIV in the past?”* with responses categorized as “Yes,” “No” or “Unknown.” Those who answered “Yes” were directed to select a specific number of STIs they had in the past: one versus two or more.

#### 3.3.5 Characteristics of drug use

Only respondents who answered as DU, CDU or CDU6 were directed to the following questions: *“What is your primary drug of choice?”* (single selection from a list of specific drugs, with an option to specify “others”), *“Have you used two or more drugs at the same time?” for* engagement in polydrug use; *“Have you ever shared needles while injecting drugs?”*; and *“Age of first drug use”* (answered with “before 18 years old” or “at 18 years old or older”).

#### 3.3.6 Age of first penetrative sex

This variable captured the age of first engagement in vaginal or anal penetrative sex and was categorized “before 18 years old” or “at 18 years old or older.”

## 4 ANALYSIS

Data analysis in this study was conducted using IBM SPSS Statistics version 25 (IBM Corp., 2017). Descriptive statistics summarized population characteristics, while inferential statistics employed univariable and multivariable logistic regression utilizing purposeful covariate selection method [30]; significant covariates at *p*<0.05 level in the univariable logistic regression model were included in the final multivariable model. The subgroup analysis of association between ART adherence and chemsex among PLHIV used similar covariate selection criteria as the main regression analysis. Variance inflation factor tests were done to ensure no multicollinearity.

## 5 RESULTS

Out of 716 survey responses initially received, 237 were excluded due to duplication (8.4%), inconsistent information (16%), non‐residency in the Philippines during data collection (6.8%) or not identifying as MSM (68.8%). The final data analysis involved 479 Filipino MSM (response rate = 66.9%).

### 5.1 General characteristics of the respondents

The age of respondents ranged from 18 to 56 years old (Median = 30; IQR = 24−34). Nearly half (47.0%) of the respondents were from Metro Manila and Luzon Regions, and 64.7% held a college degree or higher education. Moreover, 74.1% of the respondents were employed. Over half (65.1%) reported having their first penetrative sex at 18 years old or older, while 34.9% had their first penetrative sexual experience before the age of 18 (Table 1).

**Table 1:** Cross‐tabulation of demographic, sexual and HIV/STI profiles with chemsex engagement among Filipino men who have sex with men (*N* = 479)

Demographic profile	Overall	MSM not engaged in drug use nor chemsex [Non‐C/DU] (*N* = 355; 74.1%)	MSM engaged in drug use but not for chemsex [DU] (*N* = 35; 7.3%)	MSM engaged in chemsex but not recent [CDU] (*N* = 49; 10.2%)	MSM engaged in recent chemsex in the last 6 months [CDU6] (*N* = 40; 8.4%)	*p*‐value
*Age in years* ^a^						
Median (Interquartile range)	30 (25−34)	29 (25−33)	31 (27−34)	33 (27−39)	33 (29−37)	0.001^b^
*Location in the Philippines* ^c^						0.010^b^
Metro Manila and Luzon	225 (47.0)	151 (42.5)	20 (57.1)	31 (63.3)	23 (57.5)	
The rest of the Philippines	254 (53.0)	204 (57.5)	15 (42.9)	18 (36.7)	17 (42.5)	
*Education* ^c^						0.418
High school or lower	169 (35.3)	128 (36.1)	9 (25.7)	15 (30.6)	17 (42.5)	
College or higher	310 (64.7)	227 (63.9)	26 (74.3)	34 (69.4)	23 (57.5)	
*Employment status* ^c^						0.609
Employed	355 (74.1)	262 (73.8)	28 (80.0)	38 (77.6)	27 (67.5)	
Unemployed	124 (25.9)	93 (26.2)	7 (20.0)	11 (22.4)	13 (32.5)	
*Age of first penetrative sex* ^c^						0.017^b^
<18 years old	167 (34.9)	113 (31.8)	11 (31.4)	21 (42.9)	22 (55.0)	
≥18 years old or never	312 (65.1)	242 (68.2)	24 (68.6)	28 (57.1)	18 (45.0)	
*Age of first drug use* ^d^						<0.001^b^
< 18 years old	29 (6.1)		32 (91.4)	39 (79.6)	24 (60.0)	
≥ 18 years old	450 (93.9)	355 (100.0)	3 (8.6)	10 (20.4)	16 (40.0)	
*Condomless anal sex (CAS)* ^c^						<0.001^b^
≤ 6 months ago	252 (52.6)	172 (48.5)	17 (48.6)	29 (59.2)	34 (85.0)	
> 6 months or never	227 (47.4)	183 (51.5)	18 (51.4)	20 (40.5)	6 (15.0)	
*History of sexually transmitted infections (STIs)* ^d^						<0.001^b^
No STIs	246 (51.4)	196 (55.2)	20 (57.1)	18 (36.7)	12 (30.0)	
Had STIs	130 (27.1)	71 (20.0)	10 (28.6)	24 (49.0)	25 (62.5)	
Unknown	103 (21.5)	88 (24.8)	5 (14.3)	7 (14.3)	3 (7.5)	
*HIV status* ^d^						0.002^b^
HIV negative	228 (47.6)	186 (52.4)	18 (51.4)	20 (40.8)	7 (17.5)	
HIV positive	210 (43.8)	144 (40.6)	13 (37.1)	25 (51.0)	28 (70.0)	
Unknown	41 (8.6)	28 (7.9)	4 (11.5)	4 (8.2)	5 (12.5)	
*Access to antiretroviral therapy (ART)* ^d^, ^e^						0.809
No	10/210 (4.8%)	8/144 (5.6%)		1/25 (4.0%)	1/28 (3.6%)	
Yes	200/210 (95.2%)	136/144 (94.4%)	13/13 (100%)	24/25 (96.0%)	8/28 (96.4%)	
*Access to pre‐exposure prophylaxis (PrEP)* ^d^, ^e^						0.103
No	214/228 (93.9%)	172/183 (94.0%)	17/18 (94.4%)	20/20 (100%)	5/7 (71.4%)	
Yes	14/228 (6.1%)	11/183 (6.01%)	1/18 (5.6%)		2/7 (28.6%)	

^a^ Level of significance calculated using Kruskal−Wallis test.

^b^ Significant *p*‐values <0.05.

^c^ Level of significance calculated using chi‐square tests.

^d^ Level of significance calculated using Fisher's Exact tests.

^e^ Access to ART used the denominator corresponding to the total number of self‐reported HIV‐positive status, while access to PrEP corresponds to the total number of self‐reported HIV‐negative status.

Among 479 respondents, 74.1% reported never using drugs nor engaged in chemsex (non‐C/DU), 7.3% reported drug use but not for chemsex (DU), 10.2% had engaged in chemsex, but not recently (CDU) and 8.4% engaged in chemsex within the last 6 months (CDU6). Approximately 6.1% of Filipino MSM reported having first used drugs before the age of 18, while 19.8% began using drugs at 18 years old or older. Regarding self‐reported HIV status, about 47.6% were HIV negative, 43.8% were HIV positive and 8.6% reported an unknown HIV status.

There were 95.2% (200/210) reported having access to ART among respondents reporting an HIV‐positive status, while 93.9% (214/228) reported to have no access to PrEP among those reporting HIV‐negative results.

### 5.2 Characteristics of drug use among Filipino MSM engaged in chemsex

Among the 124 who reported using drugs and engaging in chemsex, the most common drugs used were methamphetamine (19.4%) and nalbuphine/Nubain (8.9%). There were 23.4% who reported polydrug use, and 12.9% engaged in needle sharing during injection.

### 5.3 Association between chemsex and self‐reported HIV status

Both univariable and multivariable regression analyses showed significant associations between chemsex and HIV status (Table 2). Compared to HIV‐negative non‐C/DU group, an HIV‐positive status was more likely associated with the CDU6 group (aOR = 5.18, 95% CI = 1.63−16.43, *p* = 0.005) and those with a history of STIs (aOR = 2.09, 95% CI = 1.28−3.41, *p* = 0.003). Moreover, having an unknown HIV status was also more likely associated with the CDU6 group (aOR = 6.88, 95% CI = 1.15−14.11, *p* = 0.035) and having an unknown STI history (aOR = 9.82, 95% CI = 3.75−25.75, *p*<0.001) but was less likely associated with recent CAS (aOR = 0.23, 95% CI = 0.10−0.53, *p*<0.001).

**Table 2:** Regression analyses assessing the association of self‐reported HIV status and chemsex engagement among Filipino MSM (*N* = 479)

	Univariable logistic regression				Multivariable logistic regression			
	HIV positive versus HIV negative		Unknown versus HIV negative		HIV positive versus HIV negative		Unknown versus HIV negative	
Characteristics	OR (95% CI)	*p*‐value	OR (95% CI)	*p*‐value	aOR (95% CI)	*p*‐value	aOR (95% CI)	*p*‐value
*Chemsex engagement*								
MSM engaged in drug use but not for chemsex [DU]	0.92 (0.44−1.94)	0.822	1.45 (0.46−4.61)	0.526	1.37 (0.62−3.02)	0.436	0.99 (0.98−1.37)	0.136
MSM engaged in chemsex but not recent [CDU]	1.59 (0.85−2.97)	0.148	1.31 (0.42−4.12)	0.647	1.69 (0.63−4.52)	0.296	0.73 (0.13−4.11)	0.722
MSM engaged in recent chemsex in the last 6 months [CDU6]	5.08 (2.16−11.97)	<0.001^a^	4.67 (1.39−15.73)	0.013^a^	5.18 (1.63−16.43)	0.005^a^	6.88 (1.15−14.11)	0.035^a^
MSM not engaged in drug use nor chemsex [Non‐C/DU]	(Ref)		(Ref)		(Ref)		(Ref)	
*Age in years*	1.01 (0.98−1.03)	0.536	1.00 (0.95−1.04)	0.829	0.98 (0.95−1.02)	0.308	1.00 (0.95−1.05)	0.924
*Location in the Philippines*								
Manila and Luzon	1.01 (0.69−1.47)	0.977	1.50 (0.77−2.92)	0.238	1.27 (0.80−2.00)	0.311	0.69 (0.31−1.57)	0.379
The rest of the country	(Ref)		(Ref)		(Ref)		(Ref)	
*Condomless anal sex (CAS)*								
≤ 6 months ago	0.81 (0.56−1.19)	0.238	0.35 (0.17−0.71)	0.004^a^	1.52 (0.98−2.36)	0.061	0.23 (0.10−0.53)	<0.001^a^
> 6 months or never	(Ref)		(Ref)		(Ref)		(Ref)	
*History of sexually transmitted infections (STIs)*								
Unknown	0.03 (0.01−0.12)	<0.001^a^	7.12 (2.97−17.07)	<0.001^a^	0.03 (0.01−0.12)	<0.001^a^	9.82 (3.75−25.75)	<0.001^a^
Had STIs	2.18 (1.38−3.43)	0.001^a^	1.73 (0.48−6.22)	0.402	2.09 (1.28−3.41)	0.003^a^	1.24 (0.32−4.77)	0.752
Never had STIs	(Ref)		(Ref)		(Ref)		(Ref)	

^a^ Significant associations at *p*‐value < 0.05.

### 5.4 Subgroup analysis: association between chemsex and ART adherence among PLHIV

Among respondents who reported HIV‐positive status and had access to ART, there were 83% (166/200) who were adherent to ART (Table 3). Among the four groups, the highest proportion of ART adherence were among non‐C/DU group (85.3%), while the highest proportion of ART non‐adherence were among the CDU6 group (29.6%). Both univariable and multivariable logistic regression analyses revealed no significant association between ART adherence and chemsex. Notably, both univariable and multivariable regression analyses showed that recent CAS within the last 6 months was negatively associated with ART adherence (OR = 0.27, 95% CI = 0.12−0.64, *p* = 0.003 and aOR = 0.29, 95% CI = 0.12−0.71, *p* = 0.007, respectively).

**Table 3:** Subgroup analysis assessing the association between adherence to antiretroviral therapy (ART) among Filipino people living with HIV who engage in chemsex (*N* = 200^a^)

				Univariable logistic regression		Multivariable logistic regression	
	Overall (*N* = 200)	Adherent to antiretroviral therapy (ART) (*n* = 166; 83%)	Non‐adherent to ART (*n* = 34; 17%)	OR (95% CI)	*p*‐value	aOR (95% CI)	*p*‐value
*Chemsex engagement*							
MSM engaged in drug use but not for chemsex [DU]	13 (6.5)	11 (84.6)	2 (15.4)	1.06 (0.22−5.12)	0.947	1.10 (0.22−5.55)	0.905
MSM engaged in chemsex but not recent [CDU]	24 (12.0)	20 (83.3)	4 (16.7)	1.16 (0.34−3.75)	0.804	1.28 (0.37−4.39)	0.694
MSM engaged in recent chemsex in the last 6 months [CDU6]	27 (13.5)	19 (70.4)	8 (29.6)	2.44 (0.94−6.33)	0.066	1.75 (0.59−5.20)	0.316
MSM not engaged in drug use nor chemsex [Non‐C/DU]	136 (68.0)	116 (85.3)	20 (14.7)	(Ref)		(Ref)	
*Age in years*							
Median (Interquartile range)	30 (26−35)	30 (26−35)	30 (27−34)	1.00 (0.94−1.05)	0.868	0.98 (0.92−1.04)	0.488
*Region in the Philippines*							
Manila and Luzon	94 (47.0)	78 (83.0)	16 (17.0)	1.00 (0.48−2.10)	0.994	1.25 (0.56−2.79)	0.583
Outside Manila	106 (53.0)	88 (83.0)	18 (17.0)	(Ref)		(Ref)	
*Condomless anal sex (CAS)*							
≤ 6 months ago	104 (52.0)	78 (75.0)	26 (25.0)	0.27 (0.12−0.64)	0.003^b^	0.29 (0.12−0.71)	0.007^b^
> 6 months or never	96 (48.0)	88 (91.7)	8 (8.3)	(Ref)		(Ref)	

^a^ There were 10/210 Filipino PLHIV who reported to have no access to ART and was removed from this regression analysis.

^b^ Significant associations at *p*‐value <0.05.

## 6 DISCUSSION

This nationwide survey conducted in the Philippines assessed chemsex as an emerging phenomenon among MSM, with methamphetamine being the primary drug of choice among Filipino MSM, and associated behaviours resonating with previous research [21, 24, 31]. Findings also reveal that 18.6% of Filipino MSM have chemsex engagement, surpassing the 13% regional average in East and South Asia [21]. Moreover, the unique approach of comparing four groups of Filipino MSM noted that the proportion using drugs in a sexual context rather than recreational. Drug use among Filipino MSM is context‐dependent as explored by previous studies [7, 23]—potentially as a form of experiential experimentation, coping mechanism, intimate connection, sexual disinhibition or performance enhancement among employees working longer shifts. This requires a combination of prevention strategies with intensified harm reduction interventions, especially for methamphetamine users.

Recent chemsex is significantly associated with an HIV‐positive status even after adjusting for significant covariates, aligning with previous research conducted in Asia [15, 28, 31, 32, 33]. Furthermore, the subgroup analysis revealed findings similar to the Manila‐based study [24]—the slightly higher proportion of ART non‐adherent MSM who were engaged in recent chemsex, and the negative association between recent CAS and ART adherence. These reaffirm the need to strengthen adherence counselling among PLHIV while addressing the underlying motivations for chemsex engagement, assessing sexual behaviour patterns and providing risk reduction strategies.

The high proportion of MSM (93.9%) without access to PrEP highlights the need for efforts towards increasing access. It is worthwhile to note, however, that there were limited PrEP guidelines in the country at the time of data gathering in 2019. The Philippines started the PrEP scaleup in 2020 through the Department of Health's interim guidelines and inclusion of PrEP in the formulary system [37, 38].

This research has limitations impacting the interpretation of findings. The cross‐sectional design prevents definitive causation between chemsex and HIV transmission. Self‐reported data collection methods may have underestimated chemsex engagement, ART adherence and self‐reported HIV status, partly due to the sensitive nature of HIV and the highly criminalized nature of drug use in the Philippines. While indicating higher chemsex prevalence among Filipino MSM than in international studies, the lack of direct comparison with national data hinders country‐specific assessment. Recruitment through social media may introduce bias by excluding MSM without internet access, affecting generalizability. ART adherence measurement may not be ideal, but using VAS in this community‐based survey was more comprehensible for respondents. The lack of timeframe only reflected respondents’ current perception towards their adherence. Additionally, lifetime STI history may be less relevant to recent sexual experiences.

## 7 CONCLUSIONS

This study examined and revealed the nuanced associations between chemsex, HIV status and ART adherence in Filipino MSM. The significant association between ART non‐adherence and recent chemsex engagement highlights the necessity for interventions promoting adherence and harm reduction within HIV care. The complex factors influencing ART adherence underscore the need to integrate person‐centred and structural interventions to support informed decision‐making among Filipino MSM engaged in chemsex.

## COMPETING INTERESTS

All authors declare no competing interests.

## AUTHORS’ CONTRIBUTIONS

RAO conceived the presented research idea. RAO conducted the data gathering, analysis, review of collected data for quality and reliability, and verification of the underlying data. RAO, N‐YK and CS contributed to the interpretation of results and led the manuscript writing and took charge of the overall direction and planning. All authors evaluated the manuscript and provided feedback and approved the final submitted manuscript.

## FUNDING

The study was supported by the United Nations Office on Drugs and Crime (UNODC)‐Philippines, and National Science and Technology Council in Taiwan (NSTC 112‐2314‐B‐006‐081‐MY3).

## Data Availability

The data that support the findings of this study are available from the corresponding author upon reasonable request.
